# Nanoengineered, magnetically guided drug delivery for tumors: A developmental study

**DOI:** 10.3389/fchem.2022.1013994

**Published:** 2022-10-04

**Authors:** Tieyu Chen, Yanyu Kou, Ruiling Zheng, Hailun Wang, Gang Liang

**Affiliations:** Pharmaceutical College, Guangxi Medical University, Nanning, China

**Keywords:** stimuli-responsive, superparamagnetic, drug delivery, daunorubicin, magnetic guided, nanocomposite

## Abstract

Fighting against tumors is an ongoing challenge in both medicinal and clinical applications. In recent years, chemotherapy, along with surgery, has significantly improved the situation to prolong life expectancy. Theoretically, and regardless of dosage, we now have drugs that are strong enough to eliminate most tumors. However, due to uncontrollable drug distribution in the body, it is difficult to increase treatment efficiency by simply increasing dosages. For this reason, the need for a drug delivery system that can release “bombs” at the target organ or tissue as precisely as possible has elicited the interest of researchers. In our work, we design and construct a silica-based nanocomposite to meet the above demand. The novel nanocomposite drug carrier can be guided to target tumors or tissue by a magnetic field, since it is constructed with superparamagnetic Fe_3_O_4_ as the core. The Fe_3_O_4_ core is clad in a mesoporous silica molecular sieve MCM-41 (represented as MS, in this article), since this MS has enormous ordered hexagonal caves providing sufficient space to hold the drug molecules. To modify the magnetically guided carriers so that they become both magnetically guided and light-responsive, benzophenone hydrazone is coupled into the molecular sieve tunnel. When a certain wavelength of light is imposed on the gating molecules, C=N double bonds vibrate and swing, causing the cavity that holds the drug molecules to change size and open the tunnels. Hence, the nanocomposite has the ability to release loaded drugs with light irradiation. The structure, loading abilities, and the size of the nanocomposite are inspected with a scanning electron microscope, a transmission electron microscope, thermogravimetry analysis, N_2_ adsorption/desorption, and dynamic light scattering The biocompatibility and *in vitro* drug molecule controlled release are tested with an SMMC-7721 cell line.

## Introduction

Hepatocellular carcinoma (HCC) is a commonly found cancer, the third largest cause of cancer-related deaths worldwide, and the largest in Southeast Asia (Han et al., 2011; Yen et al., 2020; Zheng et al., 2022). Surgery and chemotherapy are significant in the battle against hepatocellular carcinoma, saving millions of patients. Daunorubicin (DNR) is a kind of anthracycline used clinically as a chemotherapy reagent, mainly curing cancers such as acute myeloid leukemia, acute lymphoblastic leukemia, and Kaposi’s sarcoma (Deng et al., 2021; Samosir et al., 2021; Shepherd et al., 2021). However, it is not the first drug of choice for HCC because daunorubicin can be turned into daunorubicinol in the liver with serious cardiac effects. However, the situation has changed since researchers discovered that the combination of DNR with epigallocatechin-3-gallate (EGCG) and its derivatives, can reduce the production of daunorubicinol (Li et al., 2014; Zhou et al., 2020). This brings relief to those patients who have relapsed after treatment with doxorubicin, as well as to patients with multidrug-resistant liver cancer. Yet simply replacing doxorubicin with DNR, or combining the two drugs for chemotherapy, cannot alter the shortcomings in this traditional treatment for liver cancer: i.e., short half-life, inadequate lipid/water partition, and non-selective release to tumor tissue (Chang and Wang, 2018; Qiu et al., 2020). Some researchers have thus devoted themselves to the modification of novel DNR using advanced carriers. The most commonly seen proposal is liposomal modifying methods, which mainly involve improving the lipid-water partition coefficiency or bioavailability (Krauss et al., 2019; Bewersdorf et al., 2022). Some research groups have realized that the “smart-release” of the drug can offer another more important improvement, and have proposed a series of conditionally sensitive formulations (Zhang et al., 2012; Uematsu et al., 2018). Regrettably, few research groups have designed drug carriers for DNR that can achieve all the necessary requirements.

In recent years, silica-based mesoporous nanocomposites have been adopted as the most focused functional drug delivery system (DDS). The major reason is that the silanols in molecular sieves are easily modified, so that some additional properties can be easily introduced by connecting certain functional groups or molecules. Another reason is that these mesoporous materials have an extremely large surface area and pore space and are inherently suitable as supports for catalysts and some other molecules (Sun et al., 2009; Meireles et al., 2021). It is generally accepted that a successful composite for a drug should be able to load sufficient drug molecules; it should also tightly lock the drug molecules when they do not need to be released. Furthermore, the ability to deliver drug molecules to specific tissues or organs and hence minimize the possibility of wrong transport to other sites is also essential. Moreover, if the DDS can respond to certain stimuli, including light, heat, or a change in solution pH, it can then utilize this response as a “switch” to release host molecules. Most of the recent research has been devoted to meeting one or two of the requirements mentioned above, and research into a DDS that can fully achieve the ideal state, especially one suitable for DNR, has rarely been seen.

Hence, in our search, we constructed a novel “smart” nanocomposite for drug delivery. As mentioned above, the drug carrier should be able to target the morbid tissue in the first place, and therefore superparamagnetic Fe_3_O_4_ nanoballs were chosen to act as the core. The nanocomposite can be freely dispersed in the solution in the absence of an externally strong magnetic field and can aggregate to the tumor site when a strong magnetic field is applied. As the host molecule holder, the ferrous oxide was modified and coated with the MCM-41 molecular sieve. In the next step, the silica-based molecular sieve shell was further coupled with an irradiation-sensitive organic ligand which acts as “smart” caps for the MCM-41 tunnels, allowing the DDS to manifest different behavior with/without light stimulation. Additionally, biocompatibility as well as the *in vitro* release feather were tested using the MTT method. Preliminary studies show that our DDS has good biocompatibility, and its “smart” controlled release ability also achieves our desired result. According to our design, the site-specific and stimuli-responsive drug delivery system for DNR could now provide an opportunity for hepatocellular carcinoma treatment.

## Experiment details

### Materials

Daunorubicin hydrochloride (DNR∙HCl, purity>98%) was purchased from Chengdu Desite Biological Technology; benzophenone hydrazine was purchased from Shanghai Macklin, and 3-(4,5-dimethyl-2-thiazolyl)-2,5-diphenyltetrazoliumbromide (MTT) was purchased from Beijing Suolaibao Technology. PBS (phosphate buffered saline), Dulbecco’s modified Eagle’s medium (DMEM), fetal bovine serum (FBS), and trypsin (0.25% in EDTA) were purchased from Wuhan Boside Biological Technology. Penicillin and streptomycin solution with 10,000 units per ml of penicillin, 10,000 μg per ml of streptomycin, tetraethoxysilane (TEOS, AR), 3:1 poly (4-styrenesulfonic acid-co-maleic acid) sodium salt, and (3-chloropropyl) trimethoxysilane (CPTS, purity>98%), were all purchased through Sigma-Aldrich. DCM, absolute ethanol, ethylene glycol, and NH_3_∙H_2_O (28 wt%) were purchased from Qingdao Marine Chemicals. Cetyltrimethylammonium bromide (CTAB, AR), FeCl_3_∙6H_2_O (AR), and NaOH were purchased from Huai’an Kelong Chemicals. All solvents or reagents received from the companies were used directly without additional purification.

### Instrumental information

A Perkin-Elmer Spectrum-100 FT-IR spectrometer was used for IR testing via the KBr protocol. The morphology and nanocomposite sizes were obtained with a transmission electron microscope (TEM, JEM-2010, JEOL), a scanning electron microscope (SEM, S4800, Hitachi), and dynamic light scattering (DLS, Zetasizer Nano ZS, Malvern). The magnetism was studied via a sample magnetometer (MPM5-XL-5, Quantum Design). In the MTT assay, the OD value was recorded via a microplate reader (Synergy H1, Biotek, wavelength at 490 nm). XRD features were probed with an X-ray diffractometer (Rigaku Multiflex, λ = 1.5418 Ǻ). DNR concentration was tested with UV-Vis and UV-Vis-NIR spectroscopy (UV-1900i, Shimadzu). Pore size and volume were tested by N_2_ adsorption/desorption, then calculated by BJH via a Nova-l000 analyzer. DNR loading was obtained via thermogravimetric analysis (TGA, STA-6000, Perkin-Elmer) without specifications. All operations were performed at room temperature.

### Synthesis of stimuli-responsive and magnetically guided nanocomposite

For convenience, the nanocomposite in our research was denoted as BEN@MS@Fe_3_O_4_, since it included the major functions or components of the carrier and was synthesized via a five-step procedure as follows.

The magnetic-guided property of our nanocomposite was introduced by the superparamagnetic Fe_3_O_4_ core, using a procedure modified from the literature (Gao et al., 2013). 80 ml of the glycol and 2 g of PSS:MA = 3:1 were added to a beaker and stirred supersonically until the solution became clear. To the above solution was added 2.16 g FeCl_3_·6H_2_O and 6 g anhydrous sodium acetate, which was then stirred in r.t. until it became a homogeneous solution. The above mixture was then transferred into a Teflon flask and sealed in an autoclave. The reaction was initiated at 200°C in an oven for 10 h and then terminated by cooling the solution to room temperature. The black Fe_3_O_4_ product was collected with the help of a magnet, and the product was then purified with ultra-pure water and ethanol and vacuum-dried overnight.

In order to wrap the MCM-41 molecular sieve onto the Fe_3_O_4_ nanosphere smoothly, the core must first be pre-coated with amorphous silica (Mirzajani et al., 2018). The dried 0.16 g Fe_3_O_4_ was dissolved into a solution with 40 ml absolute ethanol, 2 ml water, and 2 ml ammonia. The solution was ultrasonicated for 15 min to achieve a homogeneous mixture. In 30 min, through stirring and ultrasonication, a TEOS solution (0.8 ml TEOS in 10 ml ethanol) was uniformly added to the above mixture with a syringe. Ultrasonication continued for another 60 min and the reaction quenched by removing the product, SiO_2_@Fe_3_O_4,_ with a magnet. The SiO_2_@Fe_3_O_4_ was further purified with ultra-pure water and ethanol and vacuum-dried overnight.

In the third step, the MCM-41 mesoporous molecular sieve was wrapped onto the surface of the SiO_2_@Fe_3_O_4_ nanosphere where it formed a new shell. Then 0.1 g SiO_2_@Fe_3_O_4_ was placed in 10 ml ultrapure water and ultrasonicated for 20 min, before the above suspension was added to a solution of 40 ml 0.1 mol/L of CTAB (1.46 g CTAB dissolved in 40 ml ultrapure water), and a solution of 0.6 ml NaOH (1 g NaOH in 20 ml ultrapure water), and mixed together. Under continuous ultrasonication, 50 μl of TEOS was injected into the mixture every 5 min to a total amount of 0.3 ml (Vetrivel et al., 2010). Ultrasonication continued for another 60 min, and the reaction was then quenched by removing the product with a magnet. In order to remove the CTAB used as a template while forming the molecular sieve tunnels, the product was transferred into a solution with 30 ml absolute ethanol and 4 ml concentrated HCl, and stirred continuously under ultrasonication for 1 h. The reaction was then quenched by collecting the product (denoted as MS@Fe_3_O_4_) with a magnet, before the composite was rinsed with ultra-pure water and vacuum-dried overnight.

In our nanocomposite, CPTS acts as a bridge between the molecular sieve and the gating molecule as a silane coupling reagent. 0.5 g MS@Fe_3_O_4_ was dispersed in a solution of 5 ml CPTS and 75 ml dried toluene with the help of ultrasonication, and the temperature increased to reflux for 8 h. The reaction was quenched by cooling to r.t., and the product (named CPTS@MS@Fe_3_O_4_) was rinsed with ultra-pure water and ethanol and vacuum-dried at 50°C overnight.

The CPTS@MS@Fe_3_O_4_ was coupled with a gating molecule to yield the final nanocomposite as a drug carrier. To a flask with 100 ml DMF, 0.25 g CPTS@MS@Fe_3_O_4_ and 0.5 g benzophenone hydrazone were added. The mixture was ultrasonicated for 30 min, and then the flask was heated to reflux for 8 h. The reaction was quenched by cooling to r.t., then the final product (named BEN@MS@Fe_3_O_4_) was rinsed with ultra-pure water and ethanol, and vacuum-dried at 50°C overnight.

### DNR loading and release feature of BEN@MS@Fe_3_O_4_

DNR was loaded onto the nanocomposite under 310 nm light irradiation to activate the gating ligand. To a 3 ml aquatic solution of 20 mg/ml DNR, 20 mg BEN@MS@Fe_3_O_4_ was added. After being fully dispersed under ultrasonication, it was irradiated and stirred overnight in order for the DNR to reach a loading and release balance. The carriers loaded with the drug molecule (denoted as DNR@MS@Fe_3_O_4_) were gathered with a magnet, and the nanocomposite was rinsed with ultrapure water to remove the DNR from the surface. The DNR@MS@Fe_3_O_4_ was vacuum-dried at 50°C overnight and tested via TGA. The loading rate of DNR on BEN@MS@Fe_3_O_4_ was calculated as 1.25% from the difference between DNR@MS@Fe_3_O_4_ and BEN@MS@Fe_3_O_4_ revealed in the TGA.

In order to provide data references for the following *in vitro* cell experiments, the release property of drug carriers under light/non-light stimulation was firstly tested in the pure solvent state without cells. To a 10 ml quartz vial with 5 ml PBS solution, 5 mg DNR@MS@Fe_3_O_4_ was added and continuously stirred and irritated. At 0.25, 0.5, 1, 1.5, 2, 3, 6, and 8 h, the DNR concentration was obtained via spectrometer at 480 nm. In the case of irradiation, the early release rate was more rapid. An increase of up to 39% can be seen in the results section.

### Biocompatibility of BEN@MS@Fe_3_O_4_

The cytotoxicity of BEN@MS@Fe_3_O_4_ was checked by MTT assay (Ye et al., 2020). In order to exclude the possibility that the carrier may kill hepatocellular carcinoma tissues or cells when using DNR loaded on the nanocomposite during the chemo treatment, SMMC-7721 (human hepatocarcinoma cells, purchased from the Shanghai Institute of Cell Research, Chinese Academy of Sciences) were chosen for our experiment. The cells were cultured in a full DMEM medium, which contained FBS, 89% RPMI 1640, and 1% penicillin-streptomycin. The viability of the cell line when treated with different concentrations of added BEN@MS@Fe_3_O_4_ was evaluated by MTT testing. The SMMC-7721 cell line of 180 µl was seeded into a 96-well plate when it reached the exponential growth state, then put into the incubator for another 24 h to allow the cells to adhere to the base of the wells. When around 70 to 80 percent coverage of the well area was observed under microscopy, a solution of BEN@MS@Fe_3_O_4_ (concentrations sequenced at 1.562, 3.12, 6.25, 12.5, 25.0, 50.0 μg/ml) was added to each well. After that, the 96-well plate with cell line and nanocomposite was returned to the incubator for 24 h in 5% CO_2_ at 37°C. The biocompatibility can be seen in the following: the medium with nanocomposite was replaced with MTT and incubated for another 4 h, then the incubation was quenched with 100 μl DMSO added to each of the wells. The OD reading was recorded at a wavelength of 490 nm.

$$\text{cell}\;\text{viability}\;\left(\%\right)={\left[A\right]}_{\text{test}}/\left[A\right]\text{control}\times 100\%$$

All tests were performed with 3 repeated wells and the cell viability was calculated using the above equation.

### *In vitro* release performance of DNR@MS@Fe_3_O_4_

The stimuli release performance of DNR@MS@Fe_3_O_4_ and the stimuli and magnetic-guided release were carried forward upon a 96-well plate and 6-well plate with MTT protocols. In the stimuli release performance experiment, the SMMC-7721 cell line was used and divided into stimuli and non-stimuli groups. The 96-well plate was prepared with the same procedure mentioned above in part 2.5. When the 96-well plate was ready, different amounts of DNR@MS@Fe_3_O_4_ were added to the well to reach a final concentration of 3.8 mg/ml, 3.0 mg/ml, and 2.3 mg/ml, acting as high, medium, and low dosages, respectively. Considering that 60% of the DNR will be released within 3 h from DNR@MS@Fe_3_O_4_, this is equivalent to DNR dosages of 50 µM, 40 µM, and 30 μM at 3 h. Both groups were then incubated for 6 h while the stimuli group received extra irritation. The OD reading was performed, as mentioned in part 2.5.

In the stimuli release and magnetic-guided performance experiment, a 6-well plate was chosen in order to visualize the result. 1 ml of SMMC-7721 cell-line medium solution containing, 30,000 cells, was seeded into each well of the plate when it reached the exponential growth state and placed into the incubator for another 24 h to allow the cells to adhere to the base of the well. For the magnetic-guided group, the dual functional group, and the control group, DNR@MS@Fe_3_O_4_ was added to reach a final concentration of 3.0 mg/ml, and then the plate was returned to the incubator for 6 h. In the final step, the medium with DNR@MS@Fe_3_O_4_ was replaced by MTT and incubated for another 4 h, before rejecting the MTT solution to visualize the result.

## Results and discussion

### Design and synthesis scheme of nanocomposites: BEN@MS@Fe_3_O_4_

**SCHEME 1:**
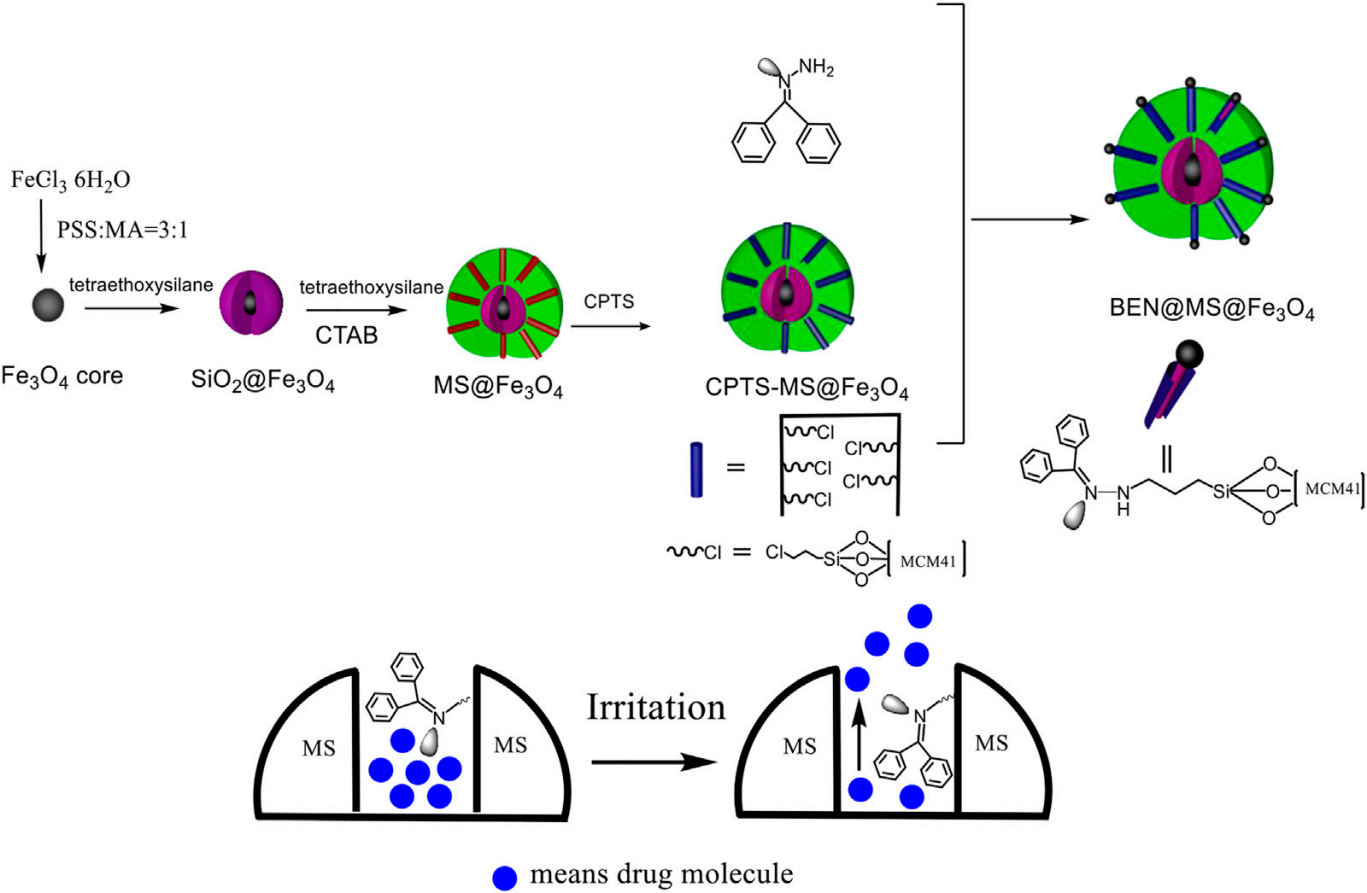
Construction of drug carrier and molecules release process.

The drug carrier in our research was firstly constructed with FeCl_3_·6H_2_O as the starting material, as shown in Scheme 1. The superparamagnetic core was formed via a hydrothermal reaction to grant the drug carrier the ability to be magnetically guided. This means the drug carrier can be easily switched from aggregation to dispersion status depending upon the presence/absence of the magnetic field and thus can be site-specific to the morbid tissues or organs. With the additional coating of a SiO_2_ crust and molecular shell, a typical core-shell nanocomposite was obtained. The mesoporous MCM-41 can offer plenty of room for host molecules. In the following steps, by linking CPTS and gating molecules, the final drug carrier was formed. The gating molecule bonded to the MCM-41 has a C=N bonding which can flip over under certain irritation. This allows the molecular sieve channels to change their size and adjust the release rate of molecules.

### Size, morphology, and distributional features of the nanocomposite

**FIGURE 1:**
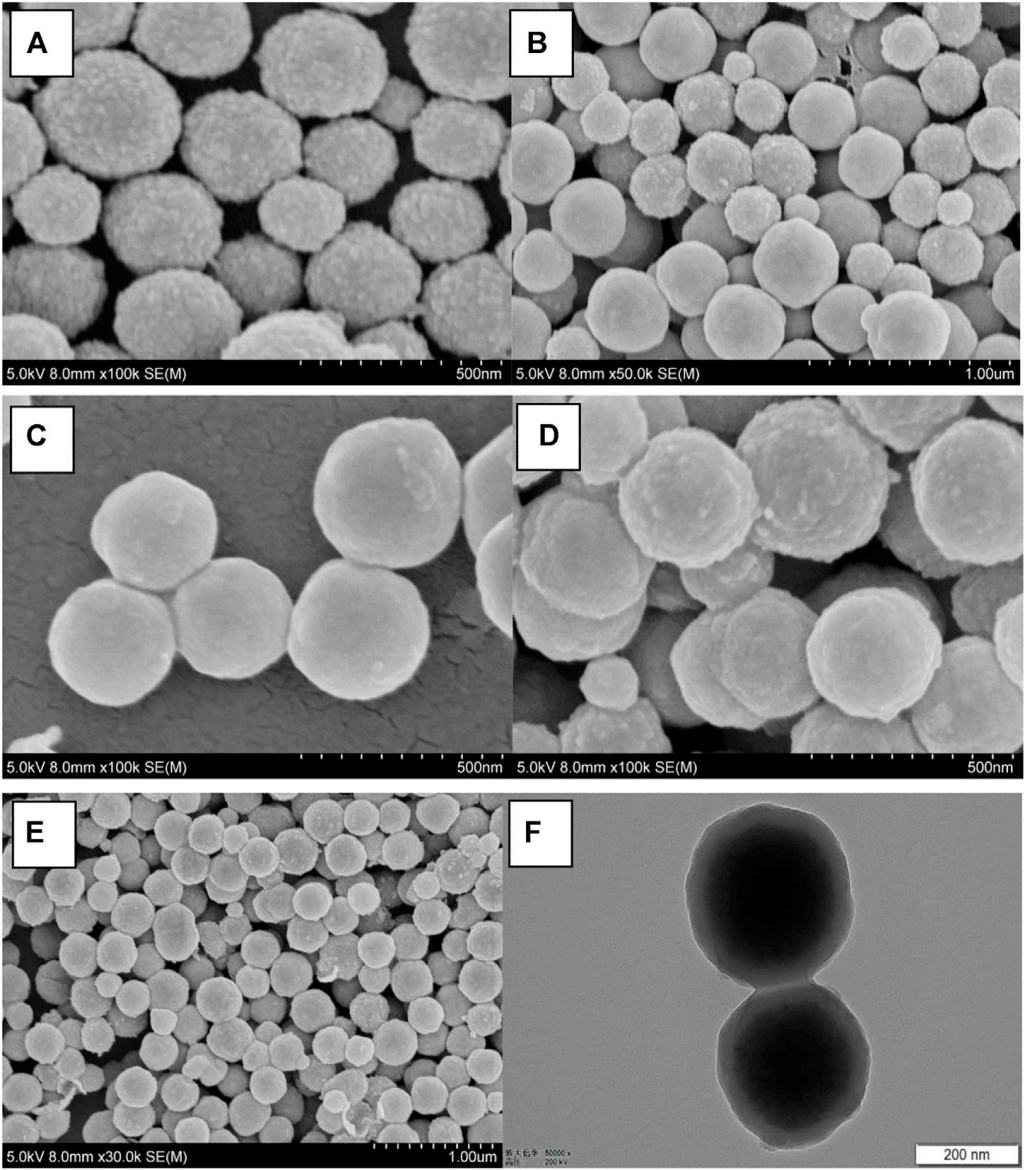
SEM micrographs of **(A)** Fe_3_O_4_ particles, **(B)** SiO_2_@Fe_3_O_4_, **(C)** MS@Fe_3_O_4_, **(D)** BEN@MS@Fe_3_O_4_, **(E)** DNR@MS@Fe_3_O_4_, and the TEM image of **(F)** BEN@MS@Fe_3_O_4_.

**FIGURE 2:**
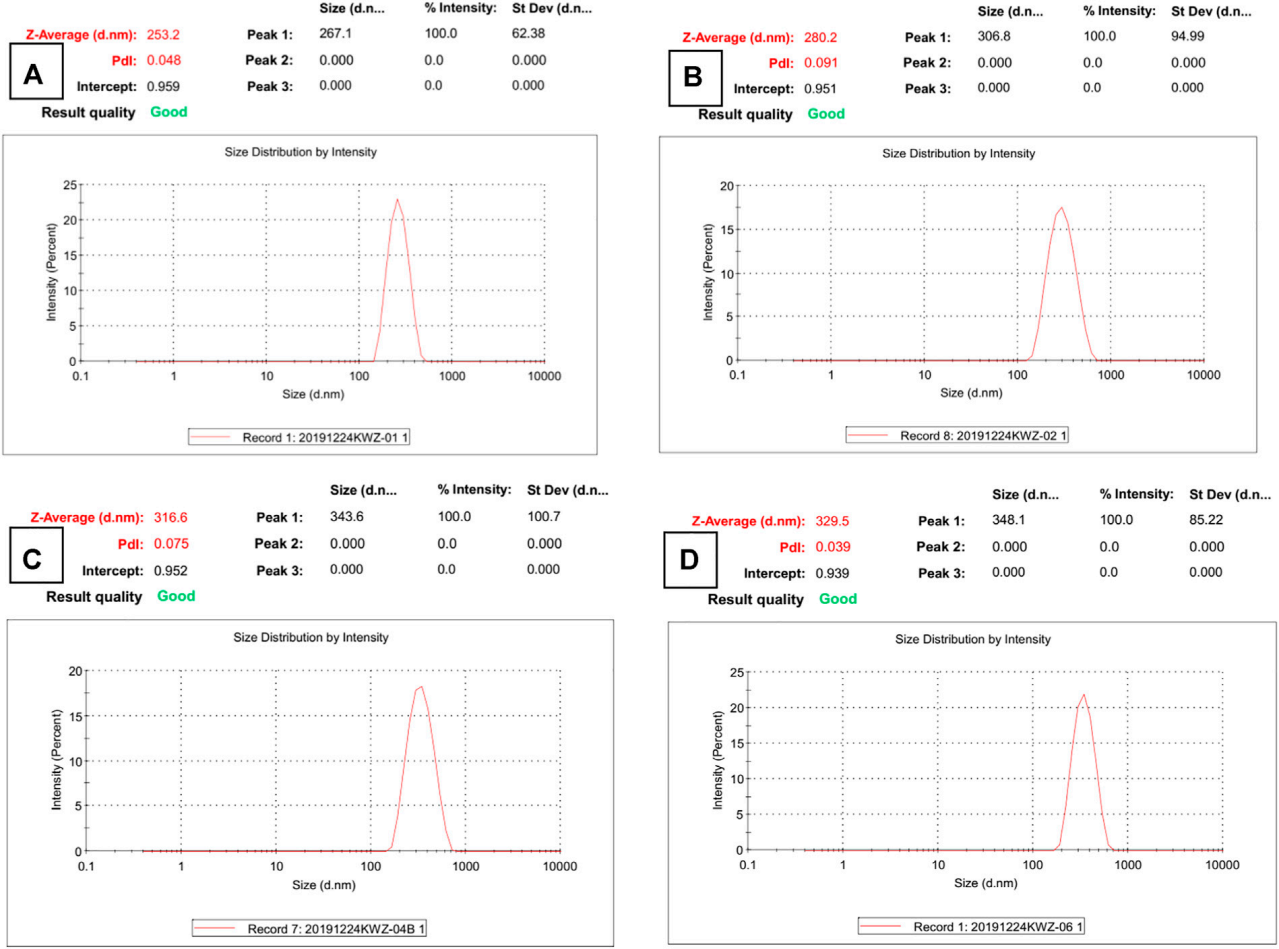
DLS results of **(A)** Fe_3_O_4_ particles, **(B)** SiO_2_@Fe_3_O_4_, **(C)** MS@Fe_3_O_4_, and **(D)** BEN@MS@Fe_3_O_4_.

The micrograph (SEM and TEM) images are shown in Figure 1, and the DLS result is shown in Figure 2. The surface of Fe_3_O_4_, as prepared by the hydrothermal reaction, is relatively rough. In fact, each large nano ball is actually made up of smaller spheres, which is a major feature of Fe_3_O_4_ prepared via hydrothermal reaction. The size of the Fe_3_O_4_ nanoparticle core prepared in the first step is about 250 nm (Figure 1A), which is consistent with the DLS testing result. The Pdi value is much less than 0.1 (Figure 2A), indicating that particle size uniformity, as well as the dispersion of the particles in the solution, is excellent, which will benefit the subsequent modification. After being wrapped in amorphous silica, the surface of the nanospheres became smoother (Figure 1B), and the average diameter of the nanospheres increased from 250 to 280 nm (Figure 2B). After further modification by molecular sieve wrapping, the diameter of the nanosphere exceeded 300 nm (Figure 2C), and the surface was smoother, close to a perfect sphere (Figure 1C). From the above, it can be inferred that the thicknesses of the amorphous silica shell and the molecular sieve shell are about 13 and 18 nm, respectively. In the subsequent reaction, both the organosilicon ligand reagent and the gating molecule were combined with the silanol groups in the molecular sieve tunnels. In the meanwhile, the DNR was mainly loaded into the MCM-41 hexagonal tunnels. Therefore, the shape and size of the subsequent nanocomposite (BEN@MS@Fe_3_O_4_, DNR@MS@Fe_3_O_4_) did not show significant change.

The final image of Figure 1F presents a perspectival view of the drug carrier BEN@MS@Fe_3_O_4_ by TEM. From the image, we can easily see the core-shell structure of the carrier. The darker sphere in the figure is the superparamagnetic core, and the lighter outer ring is the amorphous silica layer along with the molecular sieve layer. The thickness and dimensions of each component are also consistent with SEM and DLS.

### FT-IR spectrum

**FIGURE 3:**
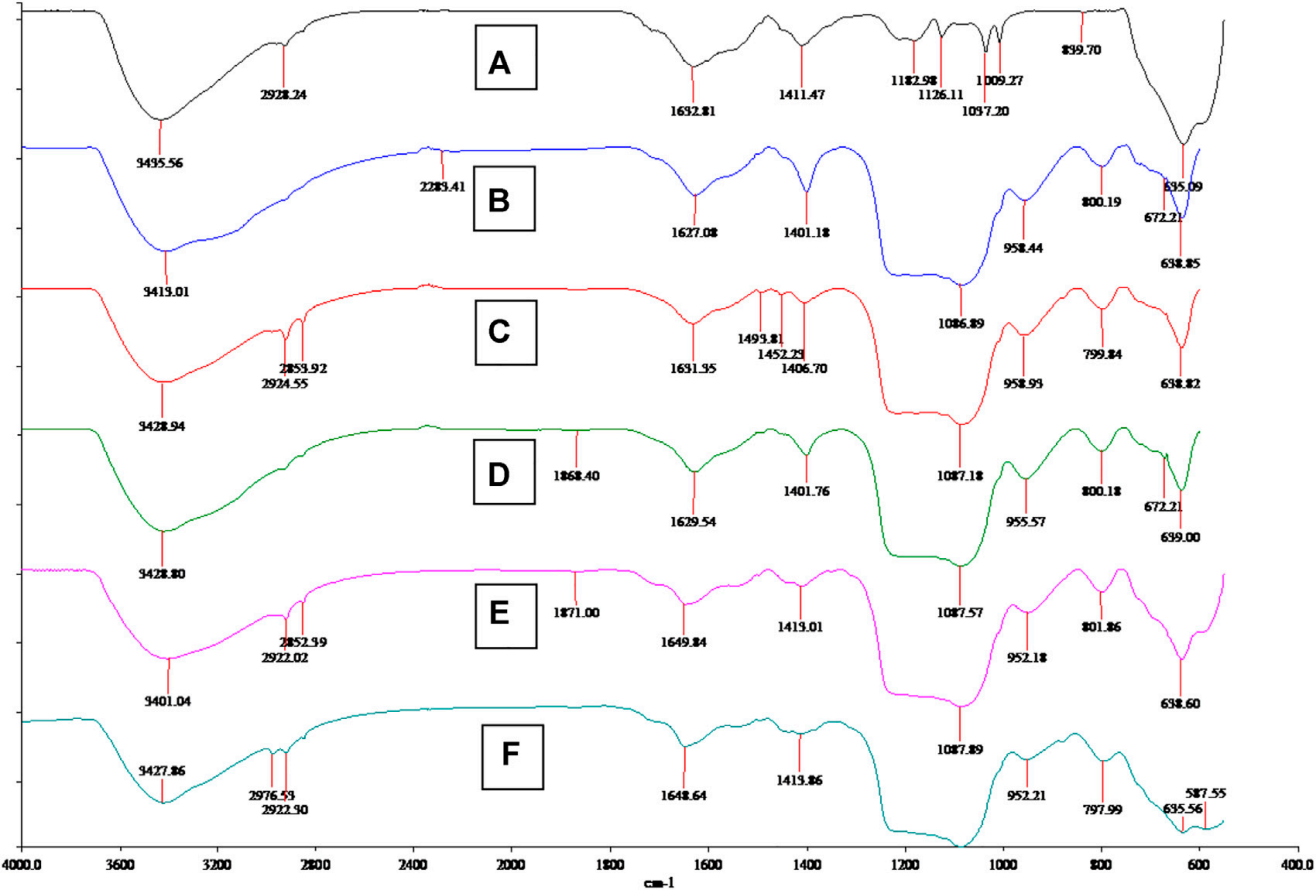
FT-IR spectrum of **(A)** Fe_3_O_4_ particles, **(B)** SiO_2_@Fe_3_O_4_, **(C)** MS@Fe_3_O_4_ (with template), **(D)** MS@Fe_3_O_4_ (without template), **(E)** CPTS@MS@Fe_3_O_4_, and **(F)** BEN@MS@Fe_3_O_4._

Besides the morphological analysis, FT-IR analysis also strongly confirmed the nanocomposite structures and organic ligand grafting step. As shown in Figure 3, the Fe_3_O_4_ spectrum is quite simple, showing Fe-O signal peaks at 3,435 and 635 cm^−1^ (Palomino et al., 2004)^.^ After SiO_2_ and molecular coating, nanocomposite (B) SiO_2_@Fe_3_O_4_, (C) (with template) MS@Fe_3_O_4_, (D) (without template) MS@Fe_3_O_4_, (E) CPTS@MS@Fe_3_O_4_, and (F) BEN@MS@Fe_3_O_4,_ all showed additional peaks at about 800 and 1,087 cm^−1^, which was caused by the Si-O vibration (Kefayati et al., 2016). The MCM-41 molecular sieve in our research was prepared with CTAB as a template, therefore before the removal of the CTAB, two obvious peaks at 2,924 and 2,853 cm^−1^ can be seen in the composite (C) (with template) MS@Fe_3_O_4_. When the CTAB was removed, the above two peaks disappeared, as shown at (D) (without template) MS@Fe_3_O_4_. The reappearance of the peaks at 2,922 and 2,852 cm^−1^ on CPTS@MS@Fe_3_O_4,_ resorted to the alkyl chain of CPTS, and an additional peak of 2,976 cm^−1^, indicated that C=N bonding had been successfully established on the final composite (Ravikumar et al., 2008).

### Nanocomposite magnetic behavior and features

**FIGURE 4:**
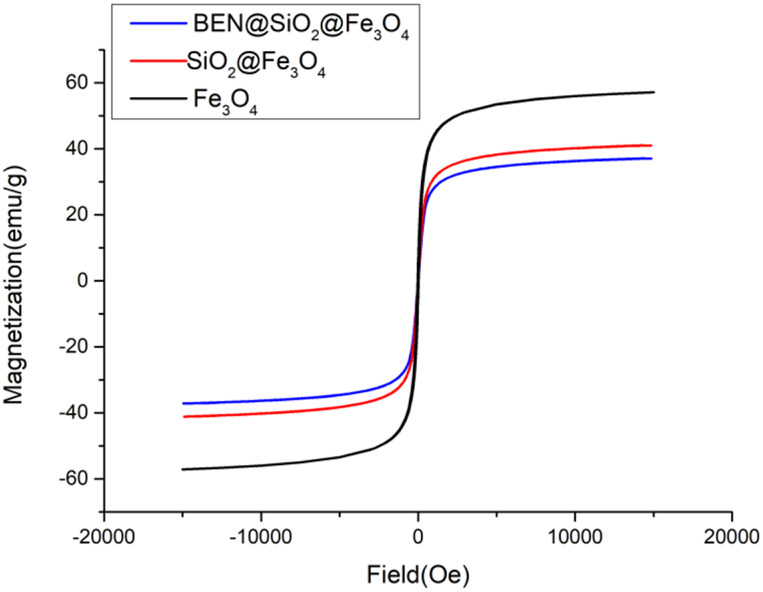
Magnetization loops for BEN@MS@Fe_3_O_4_, SiO_2_@Fe_3_O_4_, and Fe_3_O_4_.

**FIGURE 5:**
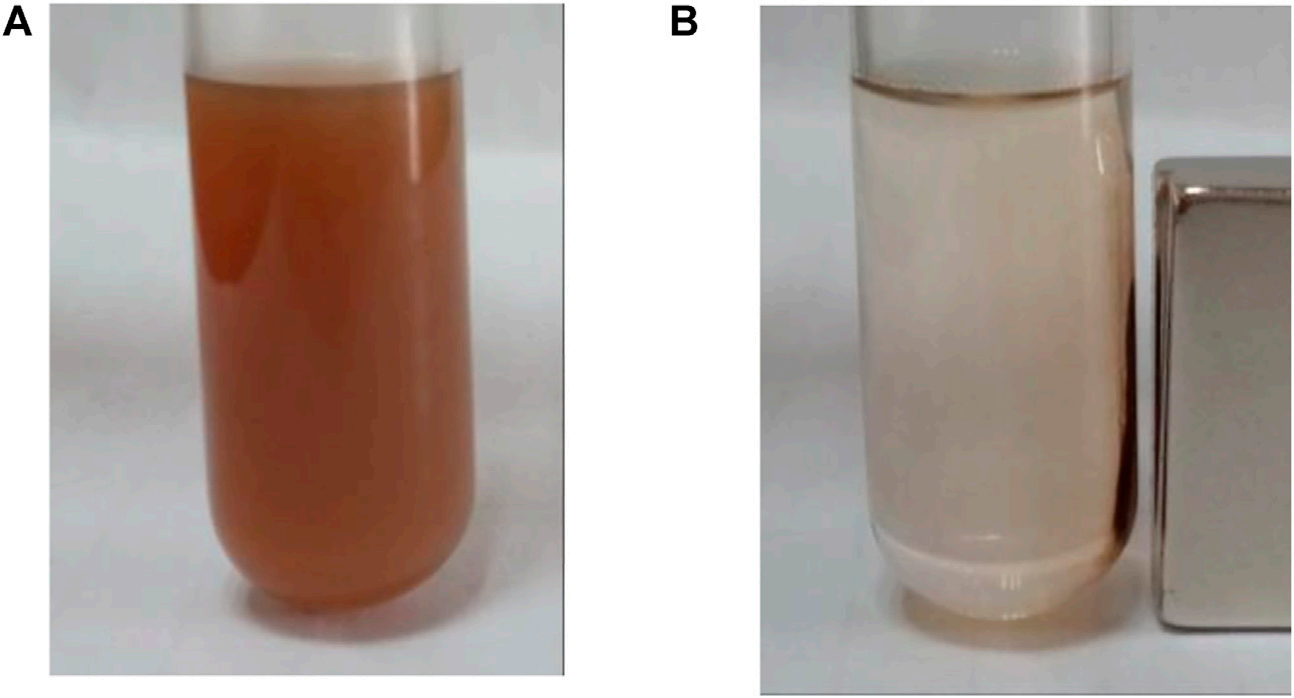
BEN@MS@Fe_3_O_4_ in ethanol solution **(A)** without magnetic field, and **(B)** with the presence of a magnet.

The carriers prepared in this study should be dispersed without a magnetic field and aggregated with a magnetic field. They must therefore be superparamagnetic. From Figure 4, it can be inferred that the nano core obtained by using the hydrothermal reaction method with PSS: MA as the surfactant is superparamagnetic with a saturation magnetization reading of 58.7 emu/g. After the coating of amorphous silica, the subsequent coating of the molecular sieve, and the coupling modification of the organic ligands, the hysteresis of SiO_2_@Fe_3_O_4_ and BEN@MS@Fe_3_O_4_ was still zero, suggesting the superparamagnetic property still existed. It is quite reasonable that the magnetic saturation dropped to 41.1 emu/g and 37.2 emu/g, respectively, since the proportion of Fe_3_O_4_ that endowed the two nanocomposites with superparamagnetic properties had been diluted. Meanwhile, in order to visualize the magnetic behavior of the nano-carrier, we tested the aggregation behavior of BEN@MS@Fe_3_O_4_ with/without a magnetic field in an ethanol solution. It can be seen from Figure 5 that the nano-carrier prepared in our research can be automatically dissolved in polar solvents and forms a stable solution. The nanocomposite can be automatically led to a specific place when a magnetic field is used, and release drugs under stimuli.

### XRD (X-ray diffraction) analysis

**FIGURE 6:**
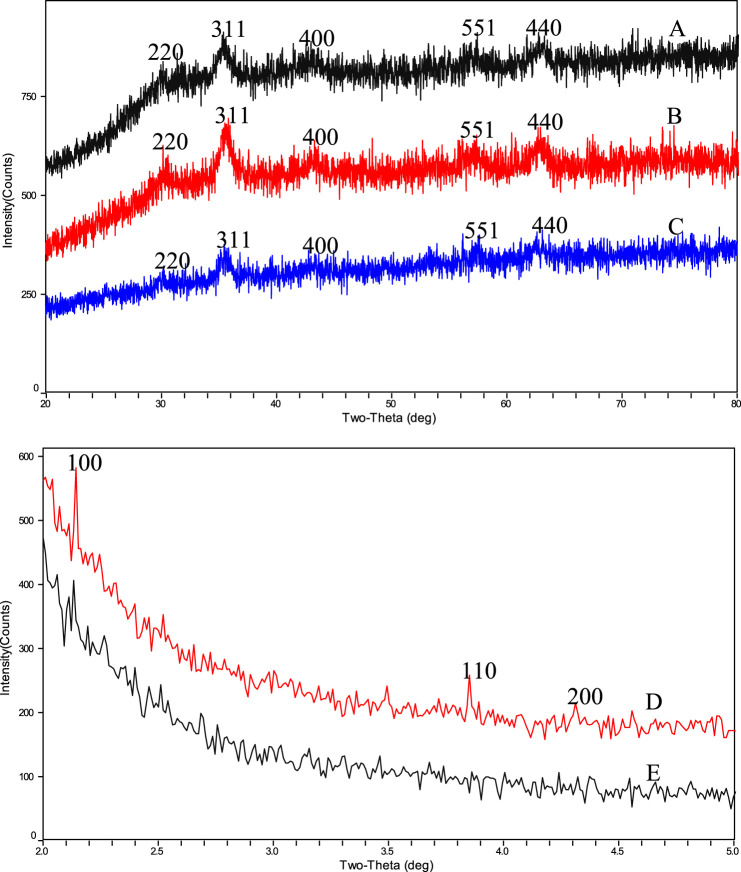
WAXRD results of **(A)** BEN@MS@Fe_3_O_4_, **(B)** MS@Fe_3_O_4_, **(C)** Fe_3_O_4_ particles and SAXRD results of **(D)** MS@Fe_3_O_4_, **(E)** BEN@MS@Fe_3_O_4_.

In order to further confirm the core structure of the drug carrier BEN@MS@Fe_3_O_4_ and its precursors, as well as the type of the outer shell, the nanocomposites were tested with wide-angle XRD (WAXRD) and small-angle XRD (SAXRD). As shown in Figure 6, the superparamagnetic core, the MS@Fe_3_O_4_, and the BEN@MS@Fe_3_O_4,_ have similar XRD patterns, and five Fe_3_O_4_ characteristic peaks marked as 220, 311, 400, 440, and 551 can be seen in all 3 samples (Gao et al., 2013). As reported in previous literature, the above peaks stand for the face center cubic structures of the superparamagnetic Fe_3_O_4_ cores. Combining the test results in the FT-IR results, we can infer that the core of the final nanocomposite BEN@MS@Fe_3_O_4_ was composed of superparamagnetic Fe_3_O_4_. In the SAXRD testing, MS@Fe_3_O_4_ and BEN@MS@Fe_3_O_4_ all showed a high Bragg reflection peak at 100, and two weak peaks at 110 and 200 (Vetrivel et al., 2010). Especially in the case of BEN@MS@Fe_3_O_4_, the two weak peaks were barely seen. This is most likely because the original regular molecular sieve channels were occupied by organic ligands and photosensitive molecules after subsequent chemical modifications. Therefore, the ordered hexagonal tunnel characteristic peaks of molecular sieves were weakened in the XRD test.

### Nitrogen adsorption–desorption isotherms of the outer shell

**FIGURE 7:**
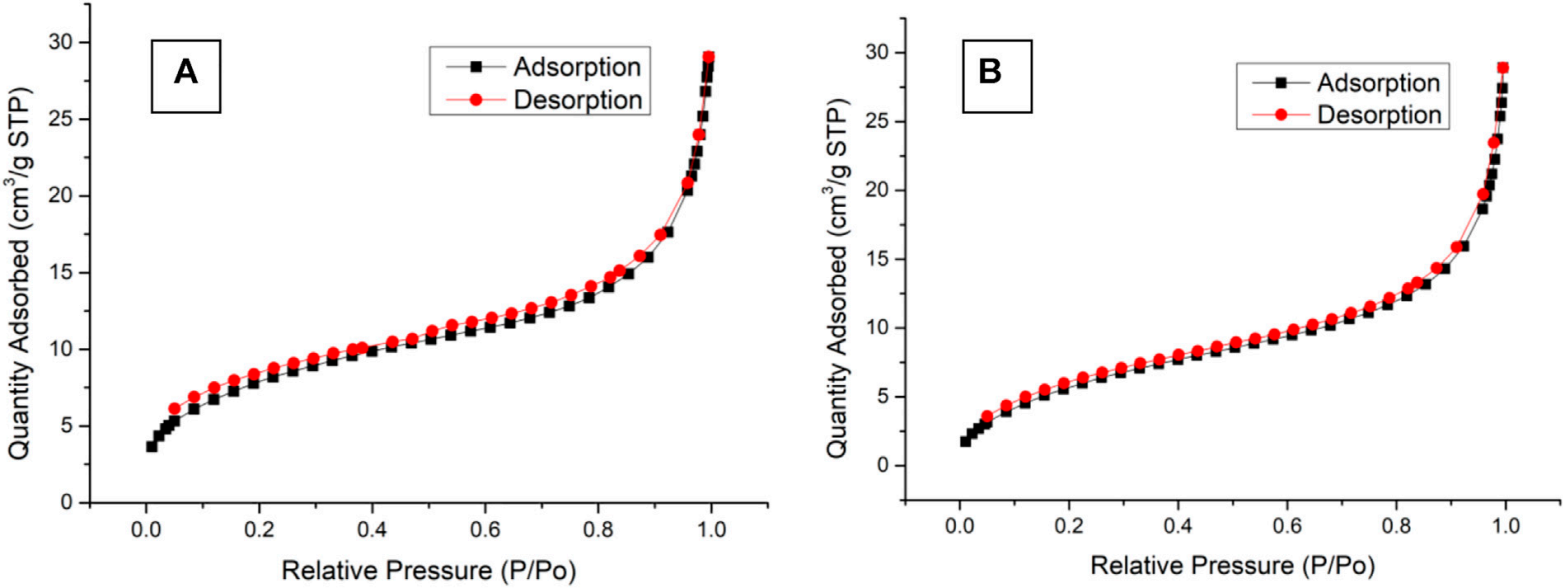
Nitrogen adsorption/desorption isotherms of **(A)** MS@Fe_3_O_4_ and **(B)** BEN@MS@Fe_3_O_4_.

The nitrogen adsorption–desorption isotherms of BEN@MS@Fe_3_O_4_ and MS@Fe_3_O_4_ are shown in Figure 7. The two samples showed the same pattern as type IV according to the literature (Huang et al., 2012). In conclusion, the MS@Fe_3_O_4_ retained its hexagonal mesophases even after template removal, organic ligand coupling, and further gating molecules modification. However, after subsequent reactions, the major feather value of the MS@Fe_3_O_4_ declined. For MS@Fe_3_O_4_, the surface area, pore diameter, and pore volume were 28.6 m^2^/g, 6.9 nm, and 0.042 cm^3^/g, respectively, and shrunk to 22.2 m^2^/g, 5.7 nm, and 0.038 cm^3^/g correspondingly. This is mainly due to the fact that the pores and tunnels in the molecular sieve had been partly occupied by organic ligands and photosensitive molecules.

### Drug loading and determination

**FIGURE 8:**
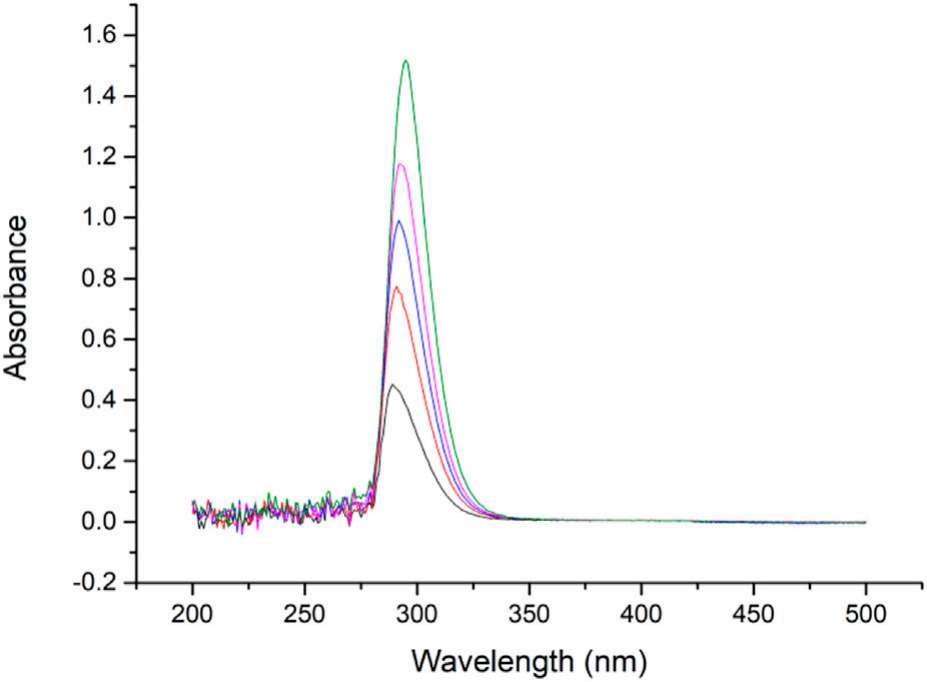
Absorbance spectrum of gating molecule (0.01–0.05 mg/ml in ethanol).

Benzophenone hydrazine connected to the MCM-41 was used for gating molecules, and can swing its C=N bond under certain irritations. Previous papers indicate that C=N bonding has a peak absorbance at approximately 300 nm (Thurston et al., 2004; Romero et al., 2015), and therefore the absorbance curve was tested. It can be seen in Figure 8 that the benzophenone hydrazine has a maximum peak at about 297 nm, hence in our research 310 nm LED was selected as an irritation source.

**FIGURE 9:**
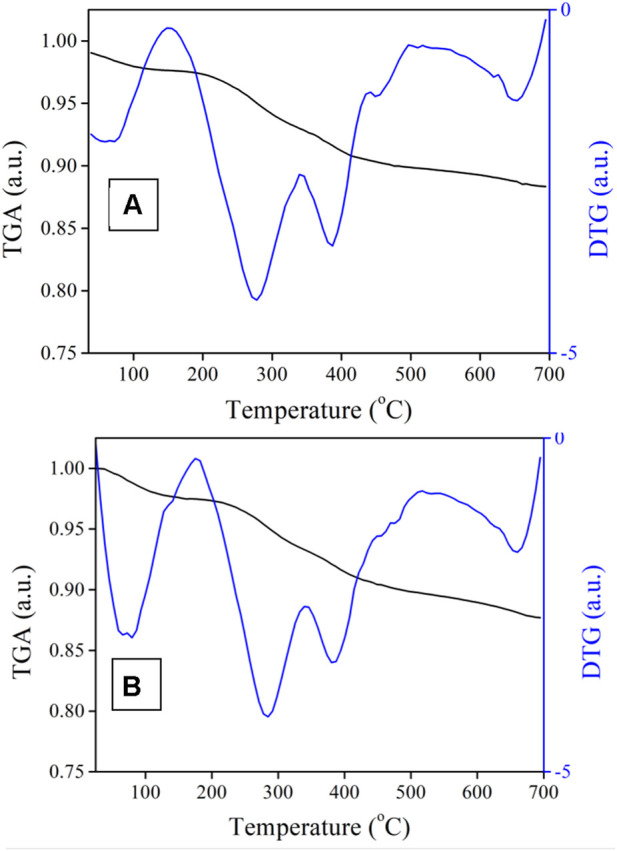
TGA of **(A)** BEN@MS@Fe_3_O_4_ and **(B)** DNR@MS@Fe_3_O_4_.

The DNR loading was studied by TGA with N_2_ as a working gas, where the temperature rising rate was 10°C per minute (Figure 9). In general, the DNR@MS@Fe_3_O_4_ lost 1.25% more weight than the BEN@MS@Fe_3_O_4_ at the final temperature; hence, we prudently concluded that the DNR loaded on the drug carrier was 1.25%.

### Cytotoxicity and analysis of the DNR@MS@Fe_3_O_4_ nanocomposite

**FIGURE 10:**
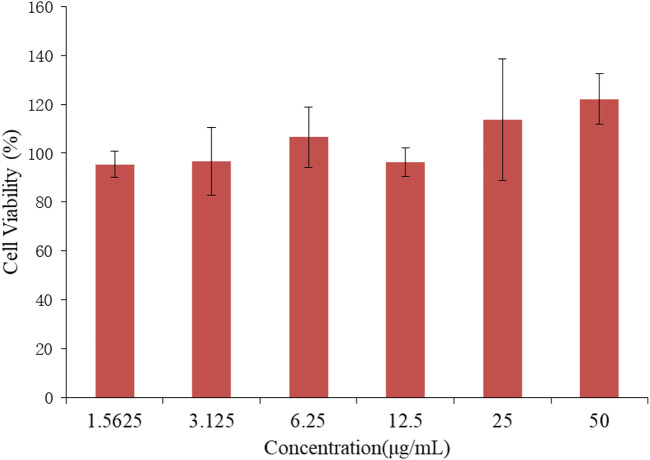
Cell viability of SMMC-7721 cell line treated with BEN@MS@Fe_3_O_4_ nanoparticle (*n* = 3).

As a carrier of drug molecules, the nanocomposite prepared in research must satisfy a fundamental property, that is, the carrier must be nontoxic and bio-safe. In this regard, BEN@MS@Fe_3_O_4_ was firstly checked for biocompatibility using the MTT method. The SMMC-7721 cell line was used to perform the testing. As is shown in Figure 10, the SMMC-7721 cell line that was exposed to the nanocomposite with a concentration of up to 50 μg/ml did not show obvious growth inhibition in this experiment. This indicates that our carrier has no cytotoxicity for the morbid tissues. We prudently believe that our drug carrier could be used as a candidate for a drug delivery system.

### *In vitro* release and performance

**FIGURE 11:**
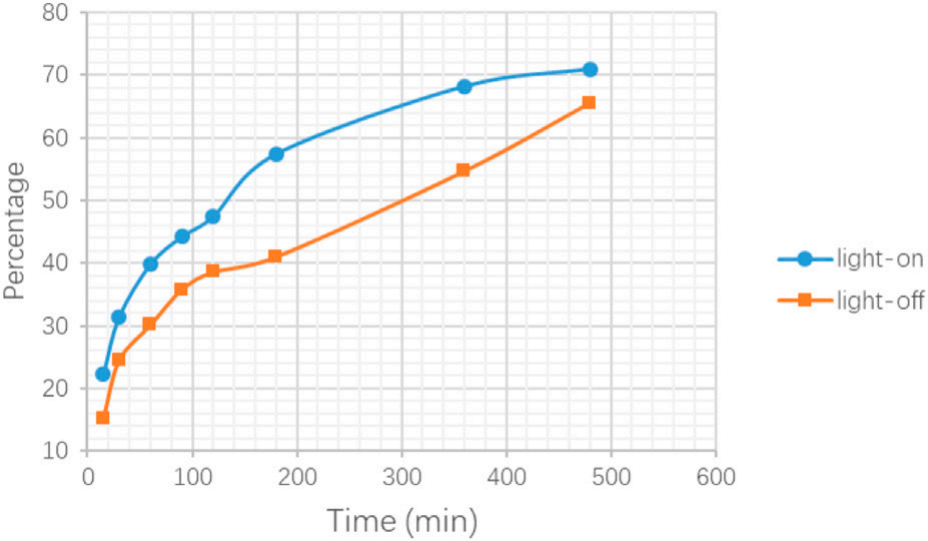
Release testing in PBS for DNR@MS@Fe_3_O_4_ with light-on and light-off.

In order to provide a reference for subsequent cell experiments, a preliminary exploration was made of the release performance upon the carrier with different conditions and different times. The result shown in Figure 11 illustrates that, under light stimulation, the drug molecules loaded on the nano-carriers were released more quickly, especially at 3 h, and the concentration increased by 39% compared with the non-light counterpart. However, the difference in drug concentration between the two experimental conditions gradually narrowed over time. On the one hand, when the drug molecules in the light-on group were rapidly released into the solution, the concentration difference between the inside and outside of the molecular sieve pores became smaller, which delayed the further release of the drug molecules. On the other hand, in the light-off counterpart, the drug molecules could also be slowly released from the molecular sieve pores through thermal motion and diffusion. Therefore, for the *in vitro* experiments, the administration time using the nano-carrier should be the time needed to reach the maximum release concentration difference between light on/off.

**FIGURE 12:**
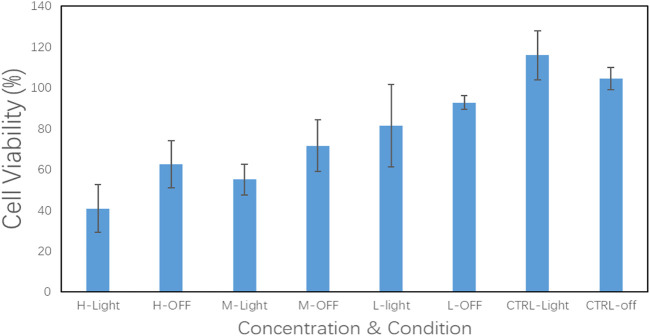
DNR@MS@Fe_3_O_4_ *in vitro* release testing upon SMMC-7721 cell line with irritation on and off (*n* = 3)

**FIGURE 13:**
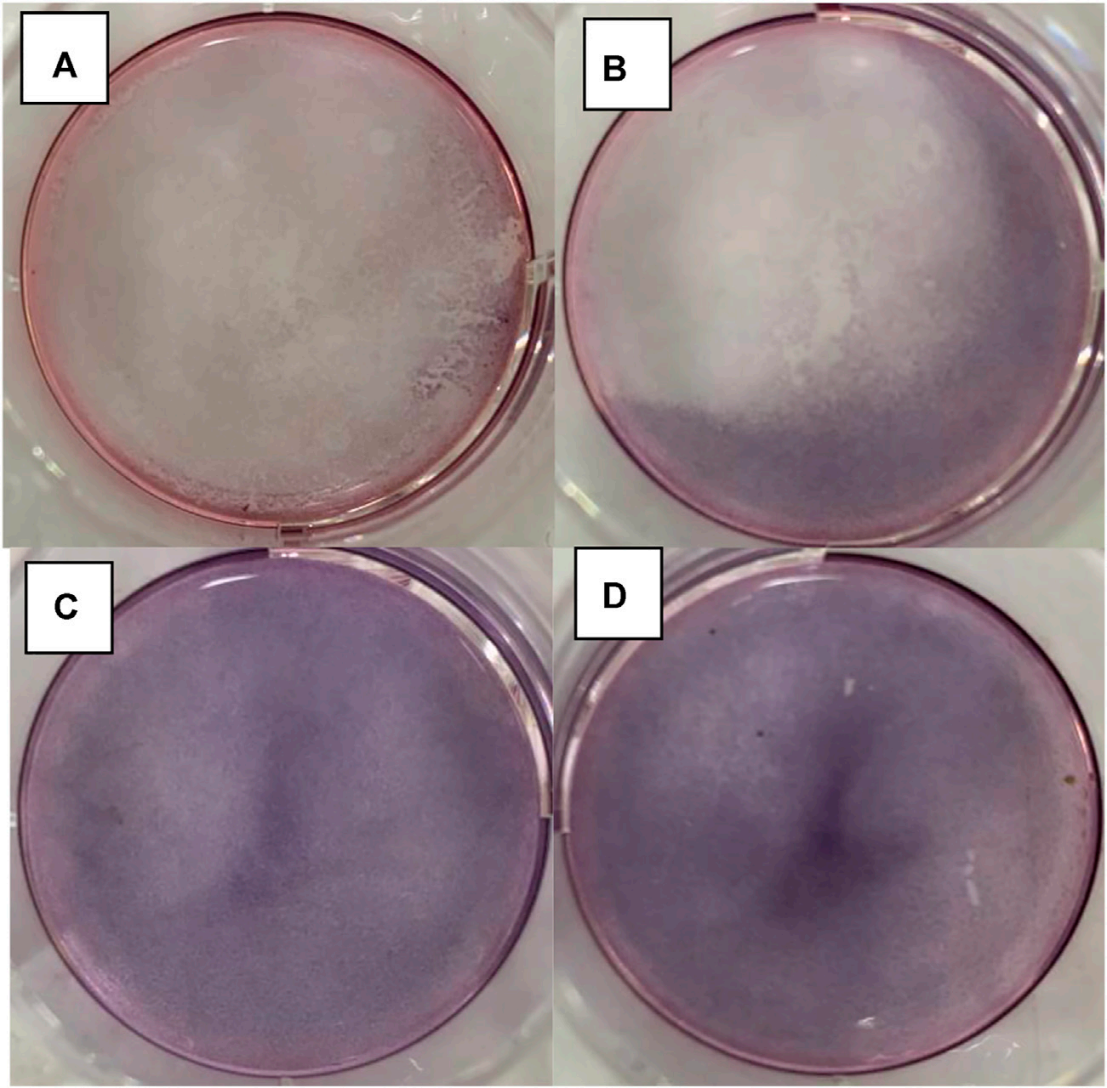
DNR@MS@Fe_3_O_4_ *in vitro* release testing upon 6-well plate method. **(A)** Light and magnetic, **(B)** magnetic only, **(C)** without light or magnetic, and **(D)** control group.

In Figure 12, the results of the *in vitro* experiments based on the 96-well plate show that under light irritation conditions, cell viability decreased obviously at each concentration. The cell line was treated with high, medium, and low dosages and the control groups are denoted as “H, M, L, and Ctrl,” respectively in Figure 12. In the meanwhile, the “Light” and “OFF” stand for the condition with and without irritation. This indicates that the drug carrier can release DNR molecules more quickly under light conditions. In the *in vitro* experiments based on the 6-well plate, the intelligent release effect of the carrier on light and magnetic guidance was more obvious. Under the same DNR@MS@Fe_3_O_4_ concentration, in the well treated by magnetic guidance and light at the same time, the cells were obviously inhibited, and the color of MTT produced was lighter. The magnetic field was applied from the left side of the well in this study, so cells near the left side were almost completely inhibited. In Figure 13A, only a few cells on the right side were left. In Figure 13B, since there was only magnetic guidance present during the test, and since no light stimulation was used, the drug concentration was not as high as in Figure 13A. However, the concentration of the drug on the left side was significantly increased, because the drug-loaded carrier was guided to the left side by the magnetic field. This made the inhibition rate on the left side significantly higher than that on the right side. In Figure 13C, since the drug was not treated with either light or a magnetic field, the drug molecules were neither stimulated to release nor locally aggregated. Hence, it is reasonable that the color depth showed as only slightly faded compared with the control group, as seen in Figure 13D.

## Conclusion

In conclusion, a novel nanocomposite with a core-shell structure was synthesized in our research. The core of the carrier was constructed for its ability to be magnetically guided, and the outer shell was designed to hold drug molecules. The detailed messages of core-shell structure in the nanocomposite were characterized by means of SEM, TEM, X-RAY, and DLS. The biocompatibility and drug loading/release properties of the carrier were preliminarily determined by using the MTT method with a 96-well plate and a 6-well plate. The results show that the nanocomposite prepared in our project has a core-shell structure. The inner core was superparamagnetic, and the outer shell was composed of SiO_2_ and molecular sieves. The 7721 cell line was used to perform the *in vitro* cytotoxicity experiment via the MTT method, and no obvious cytotoxicity was observed in the experimental result. The carriers can be aggregated under a magnetic field and can respond to irritation stimuli. Test results show that the release rate of DNR under light stimuli was significantly faster than that without light. Our nanocomposite could be a new candidate for DDS.

## Data Availability

The original contributions presented in the study are included in the article/Supplementary Material, and further inquiries can be directed to the corresponding author.
